# Using functional connectivity changes associated with cognitive fatigue to delineate a fatigue network

**DOI:** 10.1038/s41598-020-78768-3

**Published:** 2020-12-14

**Authors:** G. R. Wylie, B. Yao, H. M. Genova, M. H. Chen, J. DeLuca

**Affiliations:** 1 Kessler Foundation, Rocco Ortenzio Neuroimaging Center, 1199 Pleasant Valley Way, West Orange, NJ 07052 USA; 2 Department of Physical Medicine and Rehabilitation, Rutgers University, New Jersey Medical School, Newark, NJ 07101 USA; 3 The Department of Veterans’ Affairs, The War Related Illness and Injury Center, New Jersey Healthcare System, East Orange Campus, East Orange, NJ 07018 USA; 4 Department of Neurology, Rutgers University, New Jersey Medical School, Newark, NJ 07101 USA

**Keywords:** Motivation, Insula, Striatum

## Abstract

Cognitive fatigue, or fatigue related to mental work, is a common experience. A growing body of work using functional neuroimaging has identified several regions that appear to be related to cognitive fatigue and that potentially comprise a “fatigue network”. These include the striatum of the basal ganglia, the dorsolateral prefrontal cortex (DLPFC), the dorsal anterior cingulate cortex (dACC), the ventro-medial prefrontal cortex (vmPFC) and the anterior insula. However, no work has been conducted to assess whether the connectivity between these regions changes as a function of cognitive fatigue. We used a task-based functional neuroimaging paradigm to induce fatigue in 39 healthy individuals, regressed the signal associated with the task out of the data, and investigated how the functional connectivity between these regions changed as cognitive fatigue increased. We observed functional connectivity between these regions and other frontal regions largely decreased as cognitive fatigue increased while connectivity between these seeds and more posterior regions increased. Furthermore the striatum, the DLPFC, the insula and the vmPFC appeared to be central ‘nodes’ or hubs of the fatigue network. These findings represent the first demonstration that the functional connectivity between these areas changes as a function of cognitive fatigue.

## Introduction

Fatigue is sufficiently common in daily life as to be easily overlooked. We feel fatigued after spending a morning analyzing data, or preparing for a talk, or preparing our taxes. These are examples of cognitive fatigue, or fatigue that results from cognitive work, and although examples of cognitive fatigue abound in our daily lives, cognitive fatigue remains poorly understood^1–4^. This is partly because objective measures of cognitive performance such as response time (RT) and response accuracy have consistently failed to correlate with subjects’ reported levels of cognitive fatigue^5–7^. Thus, although individuals may perceive that they are becoming fatigued, this perception is not reflected in a reduction in behavioral performance.

More recently, studies have emerged using the tools of cognitive neuroscience to investigate cognitive fatigue. While there is a growing body of literature using EEG^8,9^ and optical imaging^10^ to investigate fatigue, here we focus on the fMRI literature. Several fatigue-related brain areas have begun to emerge, include the striatum of the basal ganglia^3,11–13^, the ventro-medial prefrontal cortex (vmPFC;^13–15^), the dorsal anterior cingulate cortex (dACC;^2,16–18^), the dorsolateral prefrontal cortex (DLPFC;^2,17,19^), and the anterior insula^2,13,17,20,21^. Research in clinical populations with severe fatigue (e.g., Multiple Sclerosis) have helped uncover the crucial role played by the cortico-striatal reward circuitry (including the striatum, the thalamus, the vmPFC, and the DLPFC) in fatigue^22^. According to this hypothesis, the amount of effort expended on a task depends on the perceived reward expected from completing the task. As effort is expended on a task, fatigue increases. Increased fatigue contributes to a decline in motivation and, in turn, task performance. Interoceptive mechanisms (i.e., awareness and monitoring of bodily functions) have also been implicated in fatigue^2,23,24^. Recent reviews on healthy individuals have integrated interoceptive and reward/motivation pathways into a motivational fatigue network consisting of the dACC, the DLPFC, the vmPFC, and the insula^2,23^. This network monitors internal bodily states, evaluates the value of pursuing the current course of action, and decides whether to continue exerting effort based on that perceived value. As fatigue increases, the relative reward ascribed to an action decreases, and additional incentives (external rewards or intrinsic motivation; e.g., monetary rewards or verbal encouragement) are required to maintain task performance.

The fatigue neuroimaging literature is limited by the heterogeneity of behavioral paradigms used to study fatigue and by the heterogeneity of the populations studied. The heterogeneity of behavioral paradigms used makes it difficult to discern if the observed neural activity reflects the experience of fatigue in general or is specific to the tasks used in the individual studies. The heterogeneity of populations studied make it difficult to discern if the observed fatigue-related neural activity is related to neurotypical fatigue or to pathological fatigue. To address these problems, the goal of the current investigation was to explore whether there is a task-independent fatigue brain network in neurotypical (healthy) individuals. This was accomplished by examining the functional connectivity among the aforementioned brain regions that have been implicated in fatigue in a neurotypical sample.

While it is known that the striatum, vmPFC, dACC, DLPFC and the insula are densely interconnected^2,25^, it is difficult to establish the extent to which these connections are sensitive to cognitive fatigue. To establish this, one would need to assess functional connectivity, a metric that is often measured at rest, in order to rule out task-induced effects. However, rest is, by its nature, not fatiguing and so it is ill suited for investigations of fatigue. We therefore reasoned that the best approach would be to use task-based fMRI using tasks that provoked cognitive fatigue. Fatigue-related changes in functional activation and connectivity provoked by task execution should occur coincidently with task-related activation and should modulate task-related activation. We reasoned that if fatigue increased across a series of runs of a given task, the task-related activation and connectivity would remain invariant (because the task was invariant), but the fatigue-related activation and connectivity would vary in proportion to the fatigue. We tested this by regressing the invariant task-related activation out of the data, and exploring the connectivity in the residuals. This allowed us to assess both the connections that were associated with fatigue, and how those connections changed as a function of increasing fatigue.

## Methods

### Subjects

Forty-eight healthy volunteers participated in this study. The behavioral data from 9 of these subjects not available due to equipment failure. Of the remaining 39 subjects, their mean age was 43.8 (± 11.7) years, their mean education was 15.4 (± 2.3) years, and 15 were women. All subjects were right handed, none had a history of neurological insult or disease, none had a history or drug or alcohol abuse, and none were on medication that would affect the BOLD signal (e.g., benzodiazepines, neuroleptics, or psycho-stimulants). All subjects provided written informed consent, in accordance with the Institutional Review Board (IRB) at Kessler Foundation and the Declaration of Helsinki, and all were compensated $100 for their time.

### Neuroimaging acquisition

Neuroimaging data collection began on a 3-T Siemens Allegra scanner (24 subjects) and was completed on a 3-T Siemens Skyra scanner (15 subjects). For this reason, a regressor for scanner was included in all group-level analyses, as has been done in previous research utilizing more than one scanner^3,26,27^. A T2*-weighted Echo Planar sequence was used to collect functional images during eight blocks (four at each of two difficulty levels), with 140 brain volume acquisitions per block (Allegra: echo time = 30 ms; repetition time = 2000 ms; field of view = 22 cm; flip angle = 80°; slice thickness = 4 mm, 32 slices, matrix = 64 × 64, in-plane resolution = 3.438 × 3.438 mm^2^; Skyra: echo time = 30 ms; repetition time = 2000 ms; field of view = 22 cm; flip angle = 90°; slice thickness = 4 mm, 32 slices, matrix = 92 × 92, in-plane resolution = 2.391 × 2.391 mm^2^). A high-resolution magnetization prepared rapid gradient echo (MPRAGE) image was also acquired (Allegra: TE = 4.38 ms; TR = 2000 ms, FOV = 220 mm; flip angle = 8°; slice thickness = 1 mm, NEX = 1, matrix = 256 × 256, in-plane resolution = 0.859 × 0.859 mm^2^; Skyra: TE = 3.43 ms; TR = 2100 ms, FOV = 256 mm; flip angle = 9°; slice thickness = 1 mm, NEX = 1, matrix = 256 × 256, in-plane resolution = 1 × 1 mm^2^), and was used to register the functional data into standard MNI space for group analysis.

### Behavioral paradigm and data

Behavioral data acquisition and stimulus presentation was administered using the E-Prime software^28^. During the fMRI scan, participants were presented with the N-back working memory task in which task difficulty was varied by presenting the 0-back condition, which places a low load on working memory, and the 2-back condition, which places a higher load on working memory. There were 4 runs of each level of the n-back task (8 runs total), with 65 trials per run. The 4 runs of each task were blocked (e.g., four runs of the 0-back task followed by four runs of the 2-back task), and the order of presentation (0-back first vs. 2-back first) was counterbalanced across subjects. During the 0-back task, participants were asked to respond each time the target letter “K” was presented on the screen, while during the 2-back task, participants were asked to respond when the target letter corresponded to the letter presented two trials prior in the sequence (e.g., R N Q N…). Letters were presented in white (Arial 72 point font) on a black background. Of the 26 letters in the English alphabet, nine were excluded to enhance the discriminability of the letters used as stimuli. The following letters were used (with equal frequency): A B C D F H J K M N P Q R S T V Z. Each letter stimulus remained on the screen for 1.5 s, followed by a 500 ms inter-trial interval (ITI), and the time between successive trials was jittered to allow for the data to be deconvolved as an event related design. The jittering was optimized using the Optseq2 program (https://surfer.nmr.mgh.harvard.edu/optseq/). The jittering was achieved by inserting between zero and six null events between successive trials. The duration of each null event was a multiple of the length of the trial (in this case, 2 s), drawn from a distribution following a power function. The majority of inter-trial intervals were 500 ms (zero null events), followed by 2.5 s (one null event) and so on. The average ITI was 1587.87 ms, and the standard deviation was 1769.7 ms. All subjects practiced both tasks prior to the scanning session.

In order to ensure comparable stimulation across subjects, the stimuli always remained on the screen for 1.5 s (that is, they were not removed when subjects responded), and each run lasted the same amount of time (260 s, or 4.33 min). The average amount of time between successive blocks was 2 min. 04 s, (S.D. = 2 min. 17 s). The behavioral data analyzed were the accuracy (the number of trials in which the correct response was made divided by the total number of trials), and the response time (RT).

### VAS-F

To evaluate the level of on-task, ‘state’ fatigue, participants were presented with a visual analogue scale of fatigue (VAS-F) before and after each block of the N-back task. Participants were asked: “How mentally fatigued are you right now?” and were asked to indicate their level of fatigue on a scale from 0 to 100, with 0 being “not fatigued at all” and 100 being “the most fatigue imaginable”. In order to mask the purpose of the study, five additional VASs were administered as well, in randomized order. These assessed happiness, sadness, pain, tension and anger.

Because VAS-F scores were obtained before and after each run, the amount of fatigue during each block was estimated by using the mean of the scores before and after the relevant block. Furthermore, because we were specifically interested in cognitive fatigue, we excluded data from runs on which subjects reported no fatigue in the analyses of functional connectivity (that is, when the VAS-F score was zero both before and after the run). Table 1 shows the number of runs on which fatigue was reported for each task. A Chi-Squared test showed the number of runs with and without fatigue was comparable across the two tasks (χ^2^(1) = 2.72, p = 0.10). Finally, because the VAS-F scores were skewed, they were transformed using the Box Cox method^29^ to ensure that assumptions of normality were not violated.

**Table 1:** Number and percentages of runs on which subjects reported no fatigue relative to runs where they reported at least some fatigue, as a function of Task (0-back vs. 2-back).

	0-Back	2-Back
Fatigue	234 (77%)	192 (71%)
No fatigue	70 (23%)	80 (29%)

### Analyses

#### Behavioral data

The VAS-F data were analyzed with a repeated measures ANOVA. The factors were Task (0-back vs. 2-back) and Rating (1st, 2nd, 3rd, 4th, 5th rating). The response time (RT) and accuracy data were each analyzed with a linear mixed effects (LME) model. The factors were Task (as above), and Run (Runs 1–4); the VAS-F scores were included as a quantitative variable, and Subject was included as a random factor.

#### Neuroimaging

The neuroimaging data was preprocessed using *fMRIPrep* 1.4.1 (Esteban et al., 2019; RRID:SCR_016216), which is based on *Nipype* 1.2.0 (Gorgolewski et al., 2011; RRID:SCR_002502).

For anatomical preprocessing, the T1-weighted (T1w) image from each subject was corrected for intensity non-uniformity (INU) with N4BiasFieldCorrection^32^, distributed with ANTs 2.2.0 (Avants, Epstein, Grossman, & Gee, 2008; RRID:SCR_004757), and used as T1w-reference throughout the workflow. The T1w-reference was then skull-stripped with a *Nipype* implementation of the antsBrainExtraction.sh workflow (from ANTs), using OASIS30ANTs as target template. Brain tissue segmentation of cerebrospinal fluid (CSF), white-matter (WM) and gray-matter (GM) was performed on the brain-extracted T1w using fast (FSL 5.0.9, RRID:SCR_002823^34^). Volume-based spatial normalization to one standard space (MNI152NLin2009cAsym) was performed through nonlinear registration with antsRegistration (ANTs 2.2.0), using brain-extracted versions of both T1w reference and the T1w template. The following template was selected for spatial normalization: *ICBM 152 Nonlinear Asymmetrical template version 2009c* [^35^, RRID:SCR_008796; TemplateFlow ID: MNI152NLin2009cAsym].

For functional data preprocessing the following preprocessing was performed on each of the eight BOLD runs of fMRI data per subject (i.e., four runs of each task). First, a reference volume and its skull-stripped version were generated using a custom methodology of *fMRIPrep*. The BOLD reference was then co-registered to the T1w reference using flirt (FSL 5.0.9^36^) with the boundary-based registration^37^ cost-function. Co-registration was configured with nine degrees of freedom to account for distortions remaining in the BOLD reference. Head-motion parameters with respect to the BOLD reference (transformation matrices, and six corresponding rotation and translation parameters) were estimated before any spatiotemporal filtering using mcflirt (FSL 5.0.9^38^). BOLD runs were slice-time corrected using 3dTshift from AFNI 20160207 (^39^, RRID:SCR_005927). The BOLD time-series (including slice-timing correction) were resampled onto their original, native space by applying a single, composite transform to correct for head-motion and susceptibility distortions. These resampled BOLD time-series will be referred to as *preprocessed BOLD in original space*, or just *preprocessed BOLD*. The BOLD time-series were resampled into standard space, generating a *preprocessed BOLD run in [‘MNI152NLin2009cAsym’] space*. First, a reference volume and its skull-stripped version were generated using a custom methodology of *fMRIPrep*. Several confounding time-series were calculated based on the *preprocessed BOLD*: framewise displacement (FD), DVARS (the spatial root mean square of the data after temporal differencing) and three region-wise global signals. FD and DVARS were calculated for each functional run, both using their implementations in *Nipype* (following the definitions by Power et al.^40^). The three global signals were extracted within the CSF, the WM, and the whole-brain masks. Additionally, a set of physiological regressors were extracted to allow for component-based noise correction (*CompCor*^41^). Principal components were estimated after high-pass filtering the *preprocessed BOLD* time-series (using a discrete cosine filter with 128 s cut-off) for the two *CompCor* variants: temporal (tCompCor) and anatomical (aCompCor). tCompCor components were then calculated from the top 5% variable voxels within a mask covering the subcortical regions. This subcortical mask was obtained by heavily eroding the brain mask, which ensured it did not include cortical GM regions. For aCompCor, components were calculated within the intersection of the aforementioned mask and the union of CSF and WM masks calculated in T1w space, after their projection to the native space of each functional run (using the inverse BOLD-to-T1w transformation). Components were also calculated separately within the WM and CSF masks. For each CompCor decomposition, the *k* components with the largest singular values were retained, such that the retained components’ time series were sufficient to explain 50 percent of variance across the nuisance mask (CSF, WM, combined, or temporal). The remaining components were dropped from consideration. The head-motion estimates calculated in the correction step were also placed within the corresponding confounds file. The confound time series derived from head motion estimates and global signals were expanded with the inclusion of temporal derivatives and quadratic terms for each^42^. Frames that exceeded a threshold of 0.5 mm FD or 1.5 standardized DVARS were annotated as motion outliers. All resamplings were performed with *a single interpolation step* by composing all the pertinent transformations (i.e. head-motion transform matrices, susceptibility distortion correction when available, and co-registrations to anatomical and output spaces). Gridded (volumetric) resamplings were performed using antsApplyTransforms (ANTs), configured with Lanczos interpolation to minimize the smoothing effects of other kernels^43^. Non-gridded (surface) resamplings were performed using mri_vol2surf (FreeSurfer).

**Figure 1:**
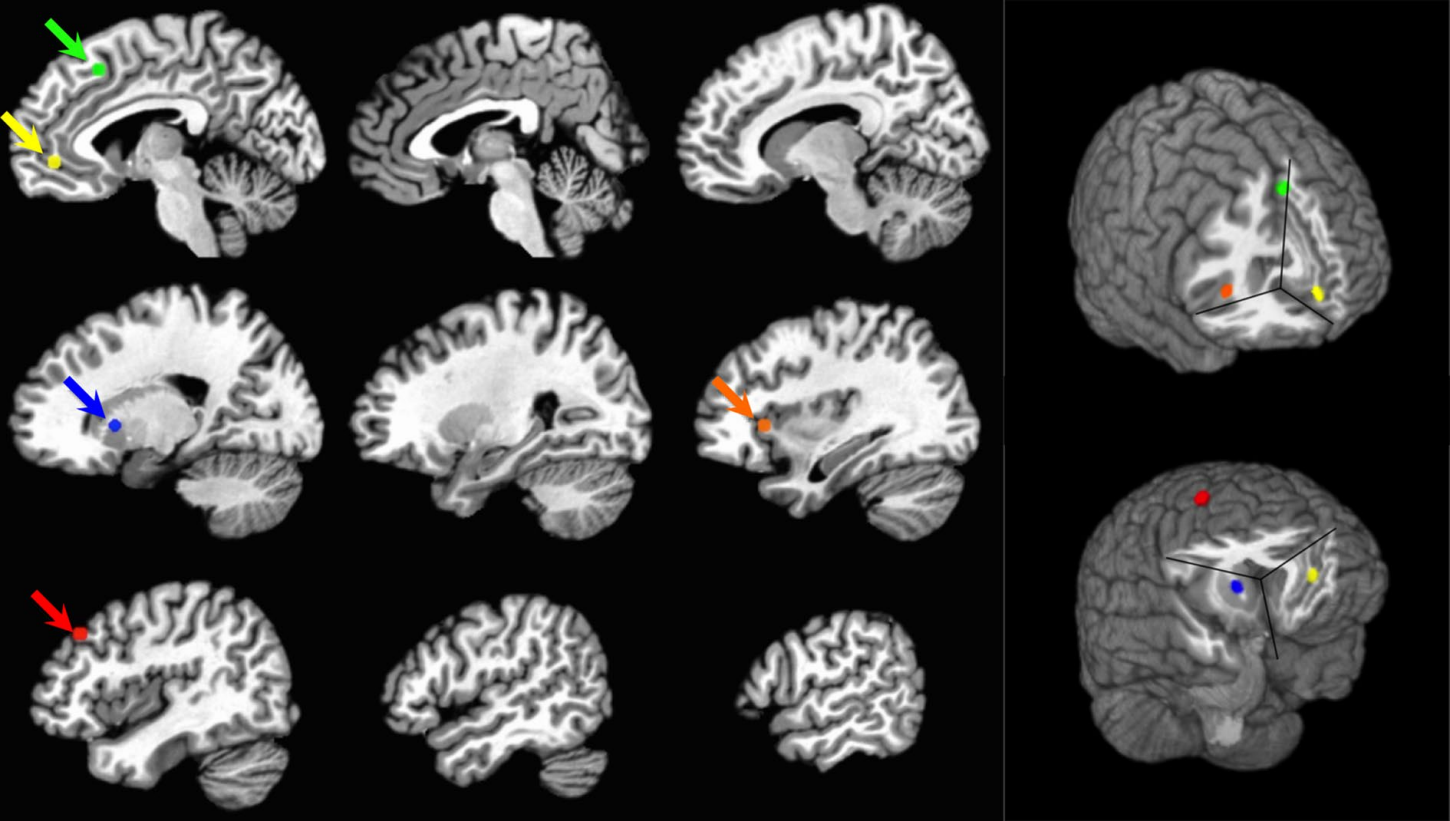
A depiction of the locations of the seeds used. On the left the seeds are shown on sagittal slices of the brain. On the right, the seeds are shown on a rendering of the brain. The seed in the dACC is shown in green; the seed in the vmPFC is shown in yellow; the seed in the striatum is shown in blue; the seed in the insula is shown in orange; and the seed in the DLPFC is shown in red.

The resulting preprocessed data from the four runs of each task were then deconvolved in a single model. In the deconvolution, signal drift was modeled with a set of basis functions; the motion parameters and their derivatives were used as regressors of no interest, as was framewise displacement. Additionally the signal from white matter, the ventricles and the global signal, and the derivatives of each of these were included as regressors of no interest. Finally, a regressor representing the onset time of each trial, convolved with a hemodynamic response function was included to model task-related activation. Importantly, the task-related activation was modeled with unit amplitude for all trials across the four runs with the result that only stable, invariant task-related activation was modeled with this regressor. The time-varying activation associated with cognitive fatigue was not modeled and was therefore included in the error term. The time-series associated with the error term was saved and used to assess connectivity between areas that have been shown to be related to cognitive fatigue (see Table 2 and Fig. 1).

**Table 2:** The locations of the seeds used in the analyses.

Location	X	Y	Z	Study
dACC	− 4	20	46	^17^
DLPFC	44	32	36	^17^
Insula	34	22	0	^17^
vmPFC	− 6	46	− 6	^44^
Striatum	18	12	0	^15^

dACC = dorsal anterior cingulate cortex; vmPFC = ventro-medial prefrontal cortex; DLPFC = dorsolateral prefrontal cortex.

For each seed (see below), the mean percent signal change was calculated within the seed for each volume of the error term time-series. The correlation between this time-series of means and every voxel in the brain was then computed, before being converted into z-scores with Fisher’s R-to-Z transformation^26^. The resulting z-score maps were entered into the group-level analysis.

#### Seeds

Five seeds were chosen for our analyses, one in each of the following locations: the dACC, the DLPFC, the Insula, the vmPFC and the striatum (see Fig. 1 and Table 2). Table 2 lists the location of the center of a 4 mm sphere used for each seed, and lists the paper upon which the X Y Z location was based.

A separate Linear Mixed Effects model (LME; 3dLME from the AFNI suite of processing tools) was used for the data from each seed. The factors in the analyses were Task (0-back vs. 2-back) and Run (runs 1–4 of each task), and Subject was included as a random factor. Furthermore, the transformed VAS-F scores (see above) were included as a quantitative variable. The inclusion of the transformed VAS-F scores allowed us to assess where in the brain connectivity covaried with fatigue.

The results of these analyses were corrected for multiple comparisons by using an individual voxel probability threshold of p < 0.001 and a cluster threshold of 13 voxels (voxel dimension = 3 × 3 × 3 mm). Monte Carlo simulations, using 3dClustSim (version AFNI_17.2.16, compile date: Sept 19, 2017) showed this combination to result in a corrected alpha level of p < 0.05. Furthermore, because of previous work implicating the striatum in cognitive fatigue^11,25^, we also calculated the cluster threshold necessary to correct for multiple comparisons in an area restricted to the nucleus accumbens, the caudate nucleus and the putamen. This calculation showed that with an individual voxel probability threshold of p < 0.001 and a cluster threshold of 3 voxels, the corrected alpha level would be p < 0.05.

#### Control analysis

In order to ensure that the connectivity patterns associated with our fatigue-related seeds were specific to the seeds chosen, we ran the same analysis using a seed in a region that has not been associated with fatigue in the literature. Because there was task-related activation at (or within millimeters of) the seed regions (see below), we chose a seed where there was robust task-related activation: primary visual cortex (see Supplementary Data). The peak activation within this area was chosen as the location for the seed.

## Results

### VAS-F

**Figure 2:**
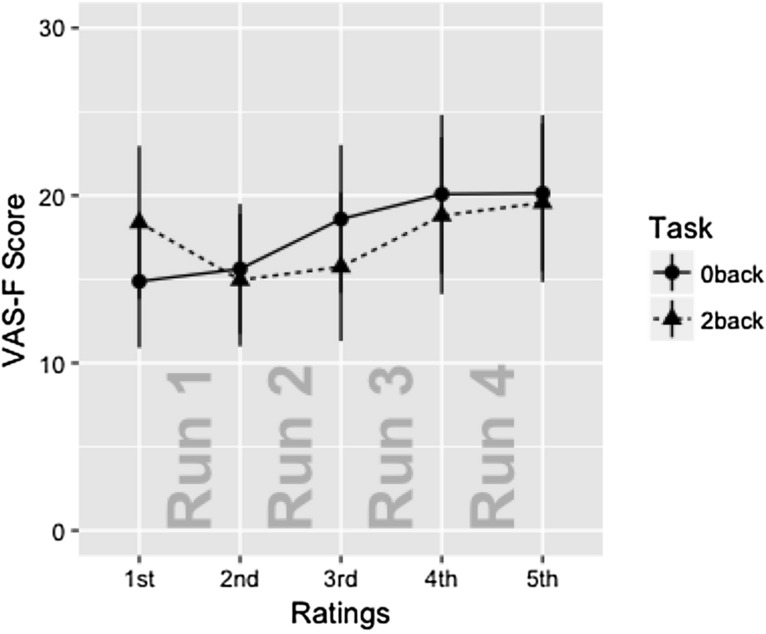
The VAS-F scores are plotted as a function of Task and Rating. Only the effect of Rating was significant. The data is separated by Task for expository purposes only (the data from the 0-back task is plotted with circles; the 2-back task is plotted with triangles). Error bars show standard error of the mean.

For the VAS-F data, the only significant effect was the effect of Rating (F(4,152) = 3.96, P < 0.005, ges = 0.006). As Fig. 2 shows, this was due to subjects responding with increasing fatigue across the 4 runs of the tasks. The means (± standard deviations) for the five ratings collapsed across Task were: 16.6 (25.2), 15.3 (23.0), 17.2 (26.1), 19.4 (27.7), 19.9 (27.7).

### Response time and accuracy results

For the RT data, there was a main effect of Task (F(1,177.3) = 139.94, p < 0.0001) and a main effect of Run (F(3,171.1) = 2.81, p = 0.04). The effect of Task resulted from subjects responding with longer latencies for the 2-back task (769.6 ms) than for the 0-back task (614.0 ms). The effect of Run resulted from subjects responding with progressively longer latencies across runs 1–3, but shorter latencies on run 4 (means for runs 1–4 were: 667.7, 704.4, 713.2, 681.9 ms). No other effects or interactions were significant.

For the accuracy data, the only significant effect was the main effect of Task (F(1,174.8) = 116.9, p < 0.0001). This resulted from subjects making more errors on the 2-back task (88.5% correct) than on the 0-back task (95.2% correct). No other effects or interactions were significant.

### Neuroimaging results

Because the VAS-F scores did not differ between the two tasks, we report the relationship between VAS-F and connectivity, irrespective of task, in the following results. For the seed placed in the dACC, there was a negative relationship between the VAS-F scores and connectivity between the dACC and frontal regions, meaning that as cognitive fatigue increased, connectivity decreased. Specifically, the coefficient was negative for the middle frontal gyrus, caudate nucleus, insula/putamen, and the middle cingulate gyrus/SMA (see Table 3). There was a positive relationship between connectivity between the dACC and more posterior regions, meaning that as cognitive fatigue increased, connectivity increased. Specifically, the coefficient was a positive for the inferior parietal lobule (bilaterally) and the angular gyrus (see Table 3).

**Table 3:** Locations showing a significant relationship between VAS-F and connectivity with the dACC, DLPFC, Insula, vmPFC and Striatum. X Y Z = the location of the voxel with peak connectivity in each cluster; Vox refers to the number of voxels in the region; VAS-F Coef refers to the coefficient of the relationship between VAS-F and the connectivity; F stat is the F statistic of the relationship. The abbreviations in brackets are the labels in the rendering. In the rendering, the color of the connections between each area and the seed represent the coefficient, ranging from − 0.074 to 0.068, shown on the right.

dACC seed						
Location	X	Y	Z	Vox	VAS-F Coef	F stat
Middle frontal gyrus [MFG]	− 23.8	9.0	34	17	− 0.052	17.12
Caudate nucleus [CN]	20.9	12.4	26	48	− 0.039	21.93
Insula/putamen [I/P]	31.2	5.5	14	15	− 0.051	23.06
Middle cingulate gyrus/SMA [MCG]	10.6	− 22.0	46	37	− 0.062	24.37
Inferior parietal lobule [IPL]	− 44.4	− 46.1	34	16	0.042	21.49
Inferior parietal lobule [IPL]	55.3	− 49.5	50	20	0.060	18.64
Angular gyrus [AG]	34.6	− 66.7	42	37	0.062	19.14
**dACC connectivity rendering**						


DLPFC seed						
Location	X	Y	Z	Vox	VAS-F Coef	F Stat
Superior frontal gyrus (including dACC and vmPFC) [SFG]	− 13.5	53.7	26	634	− 0.074	36.77
Caudate nucleus [CN]	24.3	− 4.8	22	5	0.047	13.22
Superior parietal lobule [SPL]	24.3	− 52.9	58	153	0.068	26.53
Inferior parietal lobule [IPL]	− 30.7	− 42.6	38	150	0.054	24.17
Inferior parietal lobule [IPL]	− 44.4	− 52.9	54	15	0.054	16.07
Cerebellar vermis [CB]	3.7	− 59.8	− 6	24	0.036	20.09
Cerebellum (VI) [CB]	34.6	− 52.9	− 30	14	0.047	16.98
**DLPFC connectivity rendering**						


Insular seed						
Location	X	Y	Z	Vox	VAS-F Coef	F Stat
Middle orbital gyrus [MOG]	− 34.1	50.2	− 6	17	− 0.048	19.99
Middle orbital gyrus [MOG]	31.2	46.8	− 2	100	− 0.056	25.36
Middle frontal gyrus [MFG]	− 30.7	39.9	6	24	− 0.037	21.87
Inferior frontal gyrus [IFG]	27.8	22.7	22	19	− 0.023	20.85
Anterior cingulate cortex [ACC]	0.3	39.9	2	32	− 0.064	15.89
Insula/putamen/amygdala [*]	34.6	5.5	− 14	13	− 0.068	17.04
Inferior parietal lobule [IPL]	− 47.9	− 49.5	46	14	0.041	15.87
Supra marginal gyrus/IPL [SMG]	− 61.6	− 35.7	34	34	0.052	25.75
Middle temporal gyrus [MTG]	41.5	− 1.4	− 26	21	− 0.059	23.32
**Insular connectivity rendering**						


vmPFC seed						
Location	X	Y	Z	Vox	VAS-F Coef	F Stat
Superior frontal gyrus [SFG]	27.8	70.8	− 10	69	− 0.058	25.11
Superior/middle frontal gyrus (DLPFC) [SFG]	− 20.4	29.6	46	17	0.042	18.97
Inferior frontal gyrus [IFG]	− 34.1	43.3	6	151	− 0.056	28.82
Insula [INS]	45.0	15.8	− 2	20	− 0.074	26.11
Caudate nucleus [CN]	17.5	22.7	− 10	9	− 0.057	25.44
Paracentral lobule [PCL]	0.3	− 32.3	74	16	0.033	17.54
Postcentral gyrus [PCG]	20.9	− 42.6	58	18	0.027	23.11
Posterior cingulate cortex [PCC]	0.3	− 49.5	30	48	0.053	19.79
**vmPFC connectivity rendering**						


Striatal seed						
Location	X	Y	Z	Voxels	VAS-F Coef	F stat
Inferior frontal gyrus [IFG]	− 68.5	9.0	6	51	− 0.043	19.55
Inferior frontal gyrus [IFG]	55.3	15.8	18	14	− 0.057	21.93
Insula [INS]	− 41.0	19.3	2	17	− 0.059	18.98
Inferior temporal gyrus [ITG]	45.0	− 4.8	− 30	59	− 0.045	27.36
**Striatal connectivity rendering**						

For the seed placed in the DLPFC, there was a negative relationship between the VAS-F scores and connectivity between the DLPFC and the superior frontal gyrus and a positive relationship between the DLPFC and the caudate nucleus (see Table 3). There was also a positive relationship between connectivity between the DLPFC and posterior regions: the superior and inferior parietal lobule and cerebellar regions (see Table 3).

For the seed placed in the insula, there was a negative relationship between the VAS-F scores and connectivity between the insula and several frontal regions including the middle orbital gyrus (bilaterally), the middle frontal gyrus, the inferior frontal gyrus, the ACC, and the insula/putamen (see Table 3). There was also a negative relationship between VAS-F scores and connectivity between the insula and the middle temporal gyrus. There was a positive relationship between connectivity between the insula and the inferior parietal lobule (see Table 3).

For the seed placed in the vmPFC, there was a negative relationship between the VAS-F scores and connectivity between the vmPFC and several frontal regions including the right superior frontal gyrus, the inferior frontal gyrus, the insula and the caudate nucleus (see Table 3). There was also a positive relationship between connectivity between the vmPFC and more posterior regions. Specifically, the coefficient was positive for the paracentral lobule, the postcentral gyrus and the posterior cingulate cortex (see Table 3). In addition, there was a positive relationship between connectivity between the vmPFC and left middle/superior frontal gyrus (see Table 3).

For the seed placed in the striatum, there was a negative relationship between the VAS-F scores and connectivity between the striatum and the inferior frontal gyrus (bilaterally), the insula and inferior temporal gyrus (see Table 3).

#### Task-related activation

The seeds chosen were selected based on the literature showing their involvement in fatigue. The N-back task, on the other hand, was chosen because of its ability to induce fatigue and we had no reason to expect task-related activation in the seeded regions. However, to the extent that the N-back task successfully induced fatigue, one might expect to see task-related activation in the seed regions. In an auxiliary analysis, we analyzed the task-related activation, assessing the activation associated with the two tasks (using the contrast 0-back *plus* 2-back). As shown in the Supplementary Data section, there was task-related activation at, or within millimeters, of the coordinates of each of the seeds. Furthermore, in a replication of previous research, we also compared the 2-back to the 0-back conditions (see Supplementary Data section). The results were consistent with previous findings^13,45^.

#### Control analysis

As shown in Supplementary Data Table S1, primary visual cortex showed fatigue-related connectivity with several areas. However, there was no positive relationship between VAS-F scores and connectivity with any of the areas shown in the main analyses.

## Discussion

**Figure 3:**
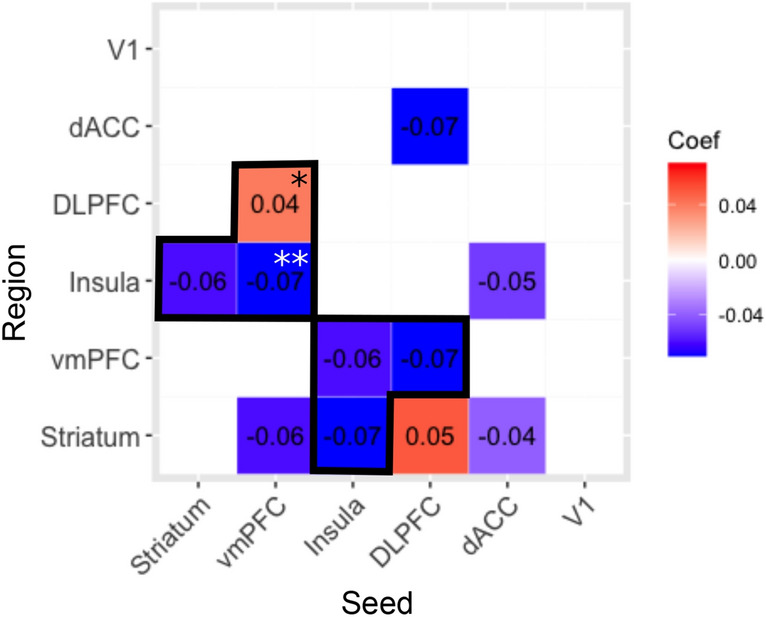
A summary of the relationships between the fatigue-related areas investigated here as well as the control area (visual cortex: V1). Blue denotes a negative relationship; red denotes a positive relationship; *denotes a relationship with the homologue of the seed; **denotes a relationship bilaterally. The relationship was reciprocal in the cells outlined in black.

Our goal was to investigate how connectivity between a prespecified set of brain areas changed as a function of cognitive fatigue in a neurotypical sample. Our results, summarized in Fig. 3, clearly support the idea that these areas comprise a “fatigue network”. While each seed region had preferential connectivity with specific brain regions, connectivity in all seeds showed fatigue-related connectivity with the striatum (see Fig. 3). Moreover, three pairs of regions showed reciprocal connectivity. These were the striatum-insula, the vmPFC-insula and the vmPFC-DLPFC. Thus, the striatum, the insula, the vmPFC and the DLPFC appear to be central ‘hubs’ in this network. This finding lends support to theories of fatigue that have highlighted the importance of these regions in fatigue^2,6,11,16,25^.

It is not entirely clear why increasing fatigue should be associated with decreasing connectivity between specific frontal areas while also being assocaited with increasing connectivity between frontal and more posterior regions. However, the hub regions identified here are part of several networks that are critical for the control of behavior: the salience network (SN, which includes the anterior insula)^46^, the reward network (RN, which includes the striatum and vmPFC)^47,48^ and the cognitive control network (CCN, which includes the DLPFC and anterior insula)^46^. One possibilty is that as subjects repeatedly perform the tasks across successive runs, cognitive resources decrease. This leads to a change in the balance between the amount of reward subjects are receiving (which is simply the intrinsic reward of doing well) and the effort required^16^, and this change is detected by the RN. In order to continue to perform the tasks despite decreasing resources, cognitive control is required, which may explain the increased connectivity between the DLPFC and parietal areas—which are part of the CCN—seen in Table 3. The observation of increased connectivity between the vmPFC and DLPFC (between the RN and the CCN) is consistent with this interpretation, since the increasae in cognitive control results from the detection of a change in the effort-reward balance. A decrease in the connectivity between the areas in the SN and the RN and CCN may be related to the finding that the SN has been associated with switching between networks^49^, and specifically between the default mode network (DMN) and the CCN^50^. If increased cognitive control is required as cognitive fatigue increases, it may be necessary to inhibit switching between the CCN and DMN and this may be reflected in reduced connectivity. More work is required to fully understand the connectivity changes observed here. Furthermore, we have established divergent validity, such that VAS-F scores did not correlate with connectivity in the primary visual cortex, which was used as a “control” region and not associated with fatigue. This strengthens our conclusions that the striatum, the insula, the vmPFC, and the DLPFC are part of a unique fatigue network.

One way forward is to investigate fatigue in clinical populations that experience high levels of fatigue. One example is Multiple Sclerosis (MS), where high levels of cognitive fatigue are often reported^6^. In a recent paper, we have shown that individuals with MS show more fatigue-related activation in more posterior brain regions than a matched group of healthy individuals^51^. This is broadly consistent with the increased connectivity between the seed regions and posterior regions shown here as fatigue increased. Furthermore, in a study of individuals with Chronic Fatigue Syndrome (CFS), it was shown that increased fatigue was related to a decrease in connectivity with the SN^52^. Our findings are not only consistent with this, but also show that decreased connectivity with the SN appears to be related to fatigue itself, and is not specific to individuals with CFS. While our results are encouraging, future work should investigate fatigue related connectivity in clinical samples to assess the extent to which fatigue related connectivity in the neurotypical sample reported here is similar to (or different from) fatigue related connectivity in clinical samples.

This study had several limitations. While our sample was relatively large, it would be reassuring to see these results replicated in another large sample. Another limiatation was that the tasks we chose induced relatively small changes in fatigue in our healthy sample. Although subjects’ VAS-F scores significantly increased over the four runs of the tasks (from 16.6 to 19.9), future work should investigate cognitive fatigue using either a task that induces more cognitive fatigue or investigate cogntive fatigue in a clinical sample where pathological fatigue is more of an issue. Additionally, though the two tasks used in this work (the 0-back and 2-back) elicited equal amounts of fatigue on the VAS-F, it is possible that the fatigue reported during the two tasks resulted from different causes. That is, during the 0-back task VAS-F scores may have reflected fatigue resulting from boredom whereas during the 2-back task VAS-F scores may have reflected fatigue resulting from diminishing cognitive resources^53,54^. Future work should distinguish between these two possiblities. Also, it should be remembered that we used a correlational approach in our analyses, and so we are unable to know whether increases in fatigue resulted in changes in connectivity or whether changes in connectivity resulted in increased fatigue.

In conclusion, by using a set of brain areas that have been shown to be related to cognitive fatigue in the literature, we have demonstrated that the striatum, the insula, the vmPFC and the DLPFC appear to be key nodes or hubs of a network of fatigue-related brain areas. We have shown not only that these areas are functionally connected, but that their connectivity changes as a function of cognitive fatigue. This represents the first demonstration of a “fatigue network” that we know of, and can serve as a foundation for investigations of cognitive fatigue in both healthy and clinical populations.

## Supplementary information

Supplementary Information
