# Supplementary material for: Using functional connectivity changes associated with cognitive fatigue to delineate a fatigue network
Source: Sci Rep. 2020 Dec 14;10:21927. doi: 10.1038/s41598-020-78768-3 (PMC7736266; doi:10.1038/s41598-020-78768-3)
Supplement: Supplementary file 1 — Supplementary Information [file 41598_2020_78768_MOESM1_ESM.doc]

**Using functional connectivity changes associated with cognitive fatigue to delineate a fatigue network**

By: Wylie, GR*^1,2,3^, Yao, B^1,2^ Genova, HM^1,2^, Chen, MH^1,2^ & DeLuca, J^1,2,4^


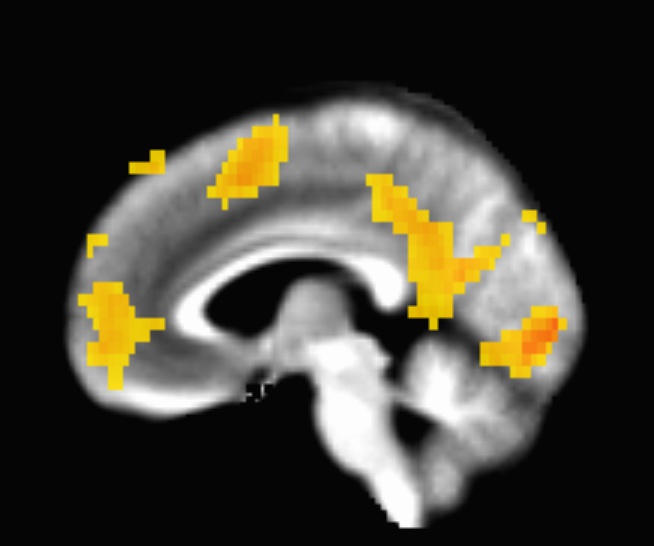


A


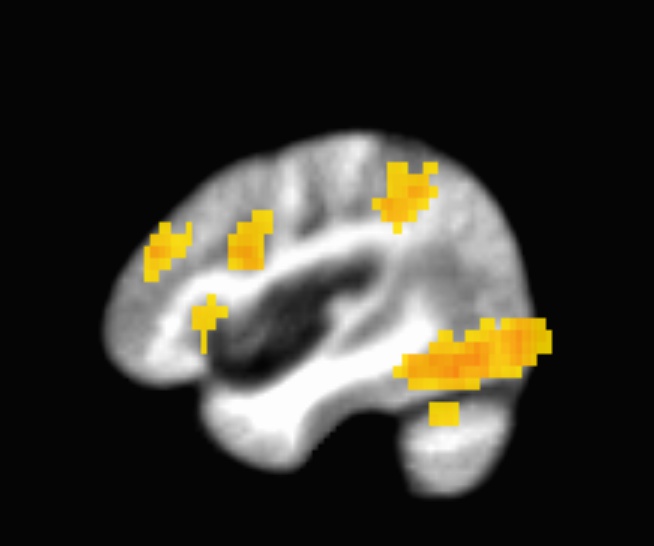


B


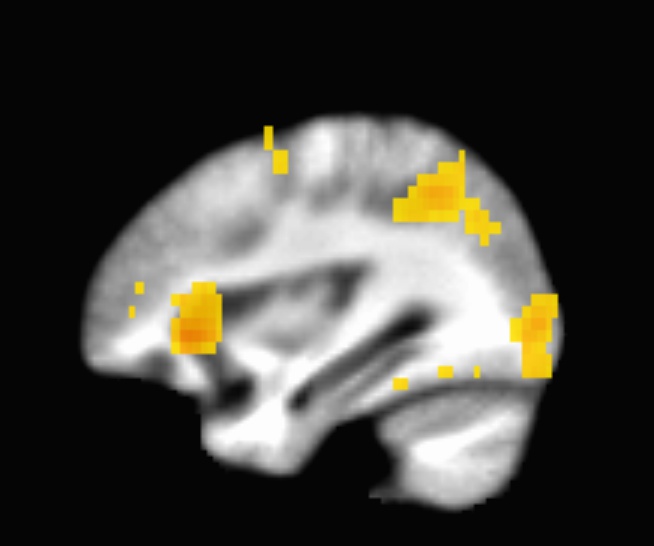


C


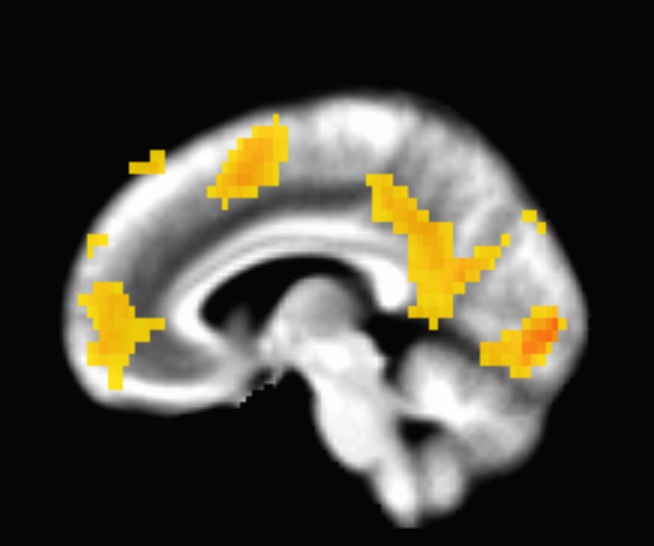


D


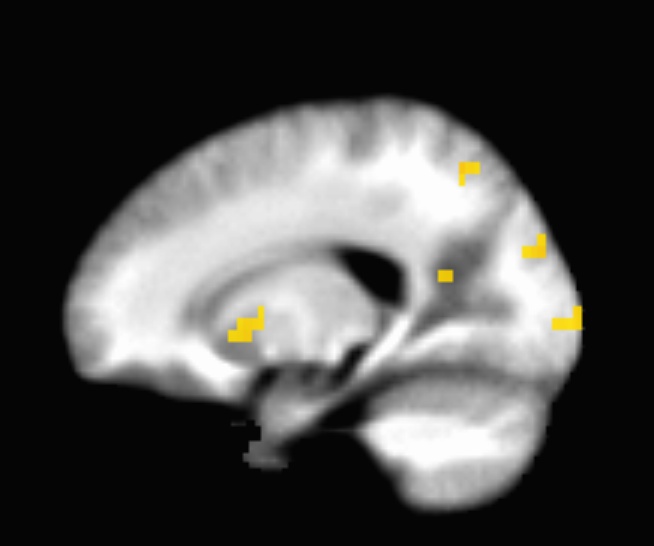


E

Figure S1. Task related activation (0-back + 2-back). The blue arrows indicate where the seeds were located for the connectivity analysis. Panel A shows the location of the dACC seed; panel B shows the location of the DLPFC seed; panel C shows the location of the seed in the insula; panel D shows the location of the seed in the vmPFC; panel E shows the location of the seed in the striatum. Panel F shows the location of the control seed, placed in primary visual cortex.


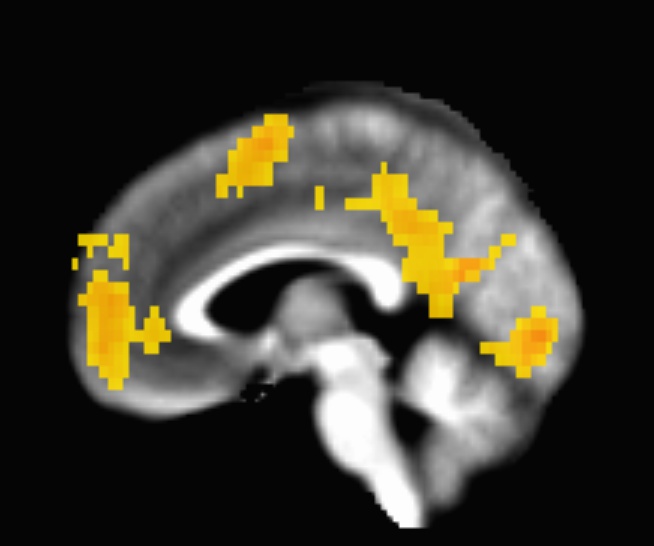


F

| **Primary Visual Cortex seed** | | | | | | |
| --- | --- | --- | --- | --- | --- | --- |
| **Location** | **X** | **Y** | **Z** | **Vox** | **VAS-F**  **Coef** | **F-stat** |
| Superior Medial Gyrus | -13.5 | 53.7 | 6 | 18 | 0.039 | 17.39 |
| Amygdala | 38.1 | -1.4 | -18 | 20 | 0.064 | 20.70 |
| Postcentral Gyrus | -68.5 | -4.8 | 34 | 18 | 0.054 | 14.35 |
| Inferior Parietal Lobule | -44.4 | -42.6 | 42 | 172 | -0.056 | 14.87 |
| Angular Gyrus | 31.2 | -63.2 | 46 | 142 | -0.057 | 14.98 |
| Middle Temporal Gyrus | -54.7 | -49.5 | -14 | 50 | -0.047 | 11.50 |
| Middle Temporal Gyrus | -41.0 | -52.9 | 10 | 50 | -0.040 | 23.21 |
| Cerebellum (Lobule VIIa) | 45.0 | -52.9 | -34 | 49 | -0.044 | 14.29 |
| Cerebellum (Lobule VIIa) | -44.4 | -73.6 | -30 | 36 | -0.047 | 25.50 |
| Cerebellum (Lobule V) | 0.3 | -63.2 | -10 | 27 | -0.056 | 11.18 |
| Cerebellum (Lobule VI) | -13.5 | -73.6 | -26 | 17 | -0.060 | 14.46 |
|  |  |  |  |  |  |  |

Table S1. Locations showing a significant relationship between VAS-F and connectivity with the Primary Visual Cortex. X Y Z = the location of the voxel with peak connectivity in each cluster; Vox refers to the number of voxels in the region; VAS-F Coef refers to the coefficient of the relationship between VAS-F and the connectivity; F stat is the F statistic of the relationship.


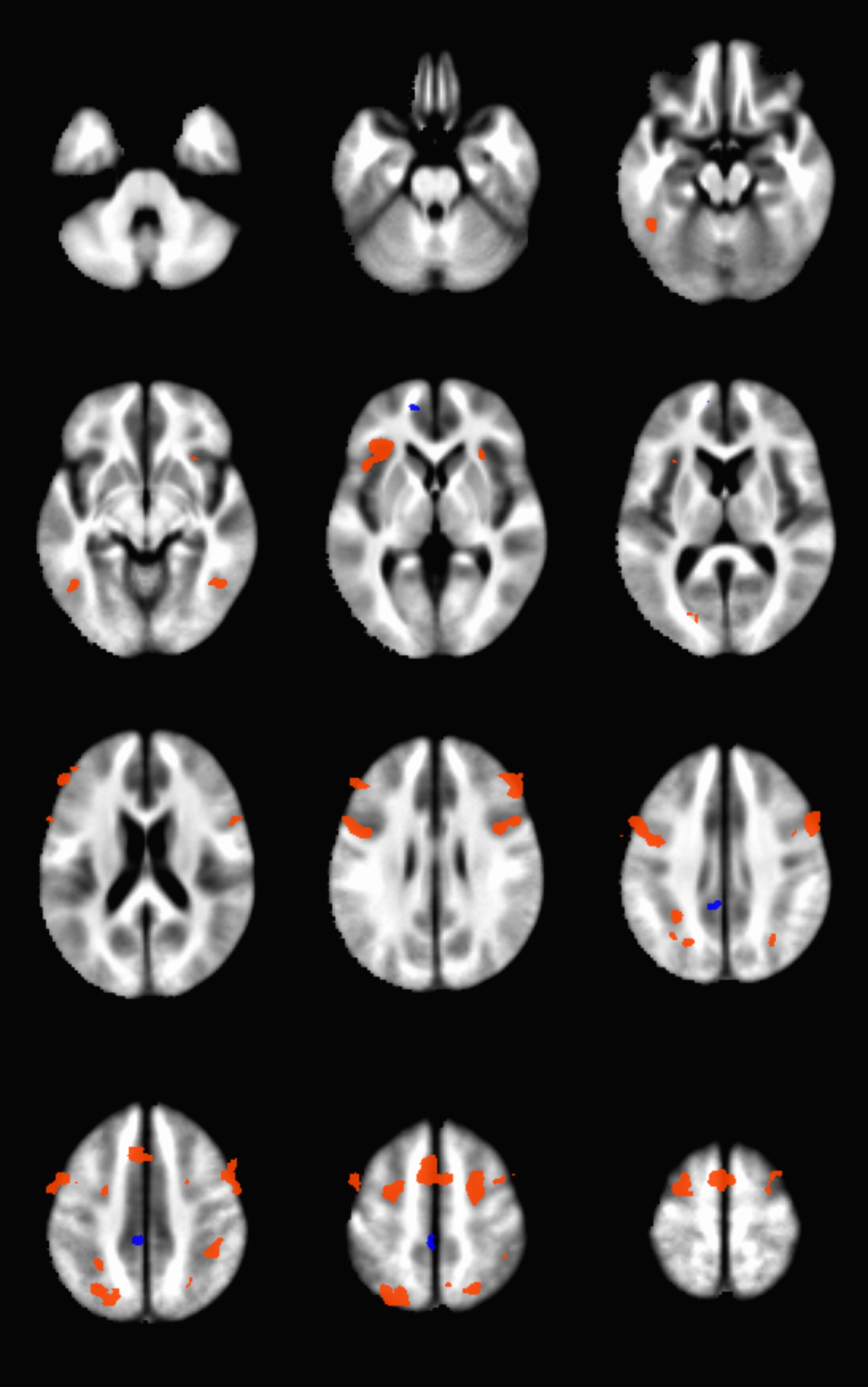


Figure S2. Difference in task related activation (2-back - 0-back). Warm colors indication areas where activation was higher during the 2-back task than the 0-back task; cool colors indicate areas where activation was higher for the 0-back task than the 2-back task.

| **N-back: 2-back minus 0-back** | | | | | |
| --- | --- | --- | --- | --- | --- |
| **Location** | **X** | **Y** | **Z** | **Vox** | **Z-stat** |
| Superior Medial Gyrus | -7.5 | 56.9 | 5.8 | 24 | -3.51 |
| Middle Frontal Gyrus | -48.2 | 6.7 | 57.7 | 230 | 3.85 |
| Middle/Inferior Frontal Gyrus | 61.8 | 18.6 | 41.7 | 351 | 3.47 |
| Middle/Inferior Frontal Gyrus | 54.6 | 40.2 | 29.7 | 73 | 3.45 |
| Insula | -41.0 | 18.6 | 1.8 | 100 | 4.46 |
| Insula | 33.1 | 23.4 | -6.2 | 22 | 3.30 |
| SMA | -2.8 | 9.1 | 57.7 | 230 | 5.22 |
| Precentral Gyrus | -38.6 | -2.9 | 73.6 | 158 | 4.08 |
| Postcentral Gyrus | -12.3 | -29.2 | 77.6 | 13 | -3.95 |
| Middle Cingulate Cortex | -2.8 | -34.0 | 45.7 | 56 | -4.04 |
| Superior Parietal Lobule | -26.7 | -74.6 | 53.7 | 193 | 4.21 |
| Superior Parietal Lobule | 21.2 | -67.4 | 61.6 | 72 | 3.37 |
| Inferior Frontal Gyrus | -55.4 | 35.4 | 21.8 | 55 | 4.00 |
| Inferior Parietal Lobule | 40.3 | -43.5 | 49.7 | 66 | 4.43 |
| Inferior Temporal Gyrus | -45.8 | -48.3 | -18.2 | 48 | 4.89 |
| Inferior Temporal Gyrus | 45.1 | -57.9 | -6.2 | 20 | 4.49 |
| Calcarine Gyrus | -21.9 | -74.6 | 5.8 | 17 | 3.97 |

Table S2. Locations showing a significant difference in activation between the 2-back and 0-back tasks. X Y Z = the location of the voxel with peak activation in each cluster; Vox refers to the number of voxels in the region; Z-stat is the Z statistic of the relationship.
